# Agro-ecological landuse transformation in oasis systems of Al Jabal Al Akhdar, northern Oman

**DOI:** 10.1038/s41598-021-85515-9

**Published:** 2021-04-08

**Authors:** Andreas Buerkert, Bryan Adam Dix, Mohamed Nasser Al Rawahi, Eva Schlecht

**Affiliations:** 1 Organic Plant Production and Agroecosystems Research in the Tropics and Subtropics (OPATS), Universität Kassel, Steinstrasse 19, 37213 Witzenhausen, Germany; 2 The Research Council (TRC), PC130 Al-Athaiba, PO Box 1422, Muscat, Sultanate of Oman; 3 Animal Husbandry in the Tropics and Subtropics, University of Kassel and Georg-August-Universität Göttingen, Steinstr. 19, 37213 Witzenhausen, Germany

**Keywords:** Ecology, Environmental sciences, Environmental social sciences

## Abstract

The millenia-old oasis systems in the Western Hajar Mountains of Northern Oman have received widespread attention as models of sustainable irrigated agriculture in hyperarid Arabia. Given Oman’s rampant urbanization, growing scarcity of water and skilled labour, we quantified chances in water use, land use, and land cover between 2007 and 2018 using a rare time-series approach of detailed GIS-based crop mapping. Results from satellite image analysis and comprehensive ground truthing showed that urban areas grew from 206 ha in 2009 to 230 ha in 2014 and 252 ha in 2018. Throughout this decade, irrigated areas in backyards and front-house gardens of the town, planted largely to tree crops and vegetables, increased from 13.5 to 23.3 ha. Between 2007 and 2018 the actively used area of the studied oasis systems declined by 2.0% and the share of perennial crops without underplanting by 5.1%, while land under agroforestry increased by 2.1% and fallow land by 3.5%. Rising water demand of the sprawling town Sayh Qatanah led to terraces of Al ‘Ayn and Ash Sharayjah now being partly irrigated with treated wastewater which accelerated the abandonment of the old settlement structures. The labour- and water use efficiency-driven transformation of the Al Jabal Al Akhdar oasis agriculture into increasingly market-oriented landuse systems questions its function as example of sustainable, bio-cultural heritage of Arabia.

## Introduction

**Figure 1:**
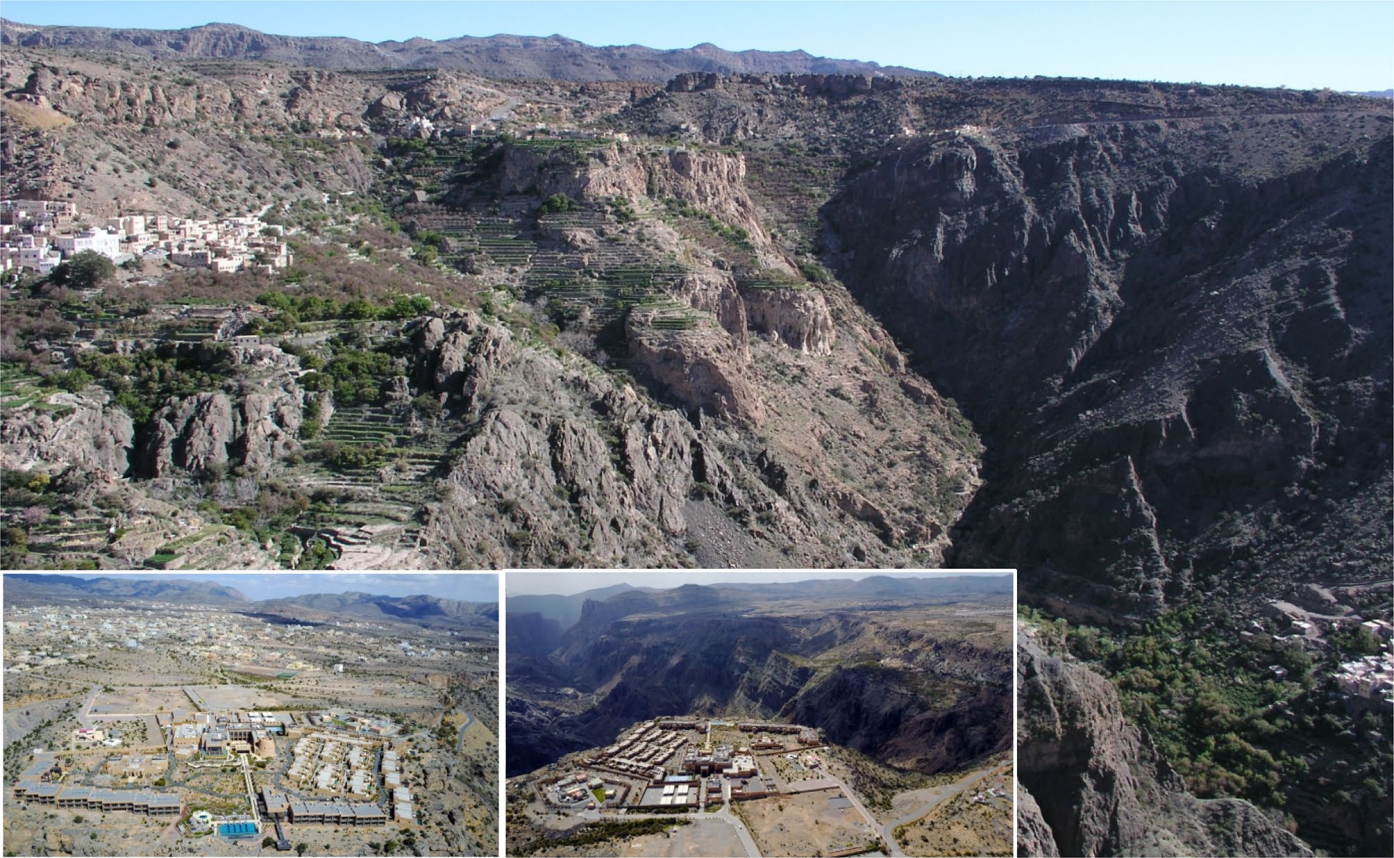
Ancient “Hanging garden” terraces of the oases of Ash Sharayjah, Al ‘Ayn and Al ‘Aqr (from left to right), the newly established town of Sayh Qatanah (background) and the oasis of Qasha’ (lower right). The two inserts show new hotel facilities on the rim of Wadi Muaydin, Al Jabal Al Akhdar, Western Hajar Mountains, Sultanate of Oman.

Most of the Sultanate of Oman’s territory is characterized by a hyperarid desert climate with irregular precipitation averaging 80–100 mm whereby 58% to 83% occur between December and April^1^. Different climate conditions prevail at its south-Eastern tip Dhofar which receives the Indian summer monsoon and in the northern Hajar Mountains, reaching nearly 3000 m a.s.l., where annual rainfall may exceed 300 mm with an exceptional peek record of 901 mm for 1997 (www.weather-and-climate.com^2^). Potential evaporation may reach around 3000 mm in the country′s interior deserts, 2100 mm on the irrigated Al Batinah coast and 1700 mm on the southern Salalah plain (Oman Water Society). The country’s highest ranges are around Jabal Shams which experience no agricultural use except for seasonal grazing by sheep and goats and the Al Jabal Al Akhdar (“Green Mountains”) which are famous for their extensive, centuries-old terrace systems, also known as “hanging gardens”^3^. Depending on the amount and distribution of rainfall, many of these *falaj*-irrigated gardens^4^ are cultivated year-round based on the intensive application of manure compost allowing nitrogen and carbon harvesting from vast grazing areas via goat and sheep herds^5, 6^. The most spectacular area comprises the oases of Wadi Muaydin, a wide dissected canyon largely made of lime- and claystones and overlooked by the Sayq Plateau. Stretching over an altitude gradient from 920 to 2000 m a.s.l. it provides many niches for endemic pants^7, 8^ and allows the cultivation of a range of tropical and Mediterranean crops at its lower and temperate cops at its higher end^9, 10^. Using the Penman–Monteith approach Luedeling and Buerkert^11^ determined the standardized reference ETsz^12, 13^ for the mountain area as ranging from 1.9 to 7.2 mm day^−1^. The area’s surprisingly large diversity reflects Oman’s rich trading history at the cross-roads of millennia of international trade^14–20^. During the last decade this watershed has also been used as an in situ laboratory to study the effects of climate change and agricultural transformation on the Arabian Peninsula^3, 10, 11^. Comparing data from 1979 to 2012, Al-Kabani et al.^21^ reported a statistical significant increase in minimum (+ 0.79 °C), mean (+ 0.27 °C), and maximum (+ 0.15 °C) temperatures per decade and a concomitant precipitation decline by − 9.42 mm. During the same period the oases settlements of Al Jabal Al Akhdar have experienced the consequences of major social-ecological transformation processes whose consequences on the landuse and land cover are poorly understood. New jobs in the secondary and tertiary sector led to rapid urbanization and brought infrastructural development to the most remote settlements; currently 85% of Oman’s population lives in urban areas and only 6.5% remain working in agriculture^22^. Also road development into formerly closed off, spectacular areas triggered a huge increase in national, regional, and international tourism which lead to the construction of vast hotel facilities^23^ (Fig. 1).

Initial surveys of Luedeling and Buerkert^11^ investigated the hydrological sustainability of the high mountain oases of Al Jabal Al Akhdar, and attributed farmer-reported water shortages to land use changes. In this context, the rapid development and subsequent water needs of the new city of Sayh Qatanah on the Sayq Plateau heading the mountain oases was identified as the greatest threat to oasis sustainability making it dependent on additional water pumped up from the lowlands. This claim was confirmed by subsequent studies of Al-Rawahi et al.^24^ and Al-Kalbani et al.^25^. The former authors particularly emphasized the surge of new major demands for irrigation water from the backyards and greenery around the spawling houses of Sayh Qatanah.

In view of the above our study was conducted to compare the mapped terrace-specific landuse of the oases of Wadi Muaydin in 2006/2007^11^ with today’s *status quo* of the agricultural areas and particularly to confront the traditional use of falaj-irrigation in terrace basins (jalba) of 1.7 to 30 m^2^ size with modern water uses on the Sayq Plateau. In particular we were interested in quantifying (i) the extent, consequences, and likely causes of putative changes in crop distribution and frequency on the traditional terraces of the “hanging gardens” and (ii) the spatial distribution and quantitative changes of irrigated land and water use in the old and new settlements.

## Materials and methods

### Agroecological setting

**Figure 2:**
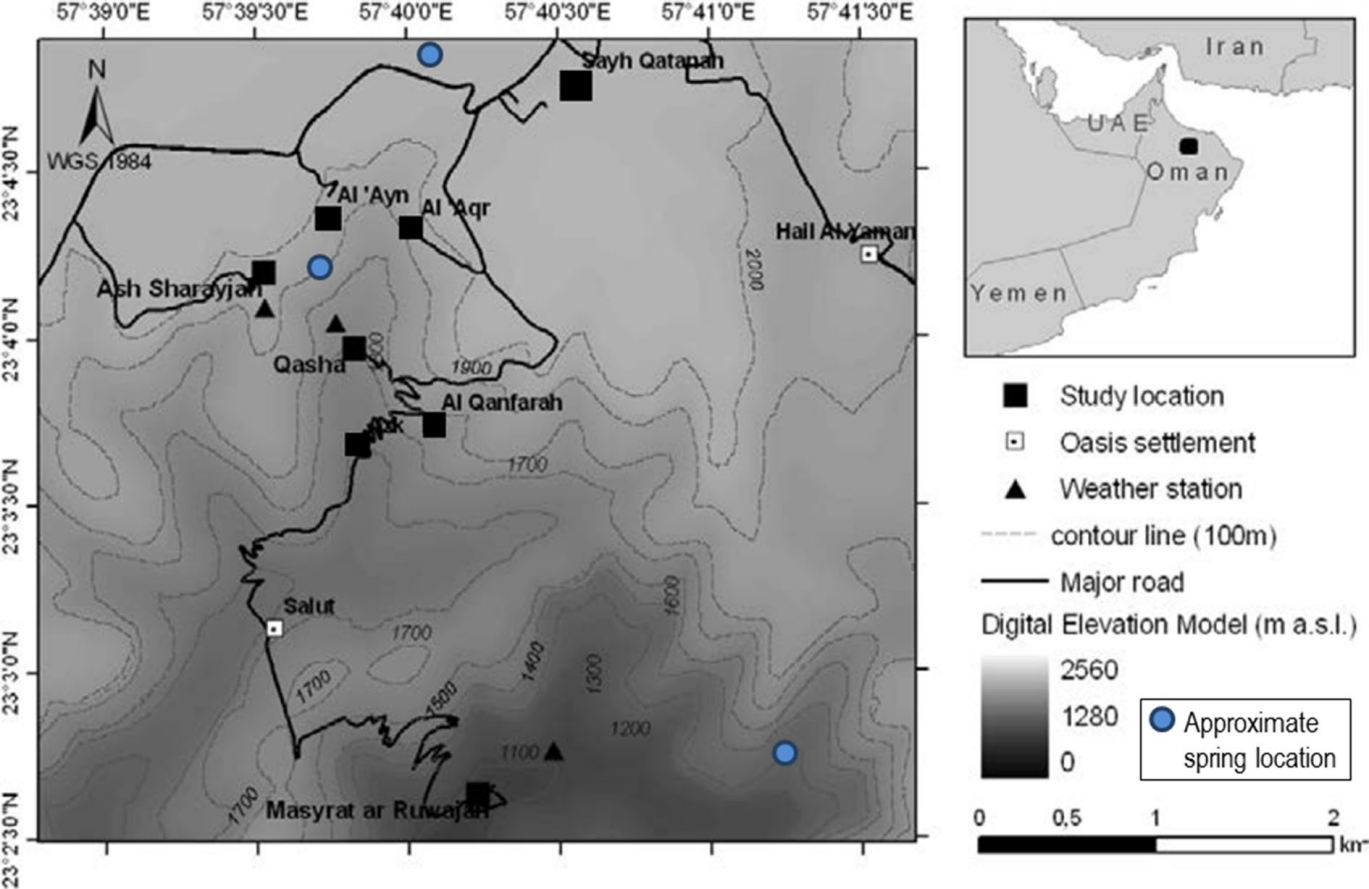
Digital elevation model of the study area on Al Jabal Al Akhdar (N-Oman; modified after Al-Rawahi et al.^3^).

The study area comprises the modern settlement of Sayh Qatanah (57° 36′ 30″ E, 23° 22′ 10″ N; 2000 m a.s.l.), a profusely growing modern town on the Sayq Plateau heading the Wadi Muaydin. It includes the oasis settlements of Al ‘Aqr (57° 39′ 58″ E, 23° 04′ 22″ N, 1950 m), Al ‘Ayn (57° 39′ 44″ E, 23° 04′ 22″ N, 1900 m) and Ash Sharayjah (57° 39′ 30″ E, 23° 04′ 10″ N, 1900 m) which are interconnected by a major *falaj* system. Below these is the settlement of Qasha’ (57° 39′ 50″ E, 23° 04′ 00″ N, 1640 m) connected to two very small settlements known as Al Qanfarah (1700 m) and Salut (1530 m). At the bottom of the watershed lies Masayrat ar Ruwajah (57° 40′ 13″ E, 23° 02′ 37″ N, 1030 m; Fig. 2). For centuries the entire watershed was irrigated by three major springs feeding two *ayni-aflaj* systems^26, 27^. The first one originates between Al ‘Aqr and Al ‘Ayn, the second one at Ash Sharayjah and their third one in the backhills of Masayrat ar Ruwajah to which it is connected by a 2 km long falaj. Recently an increasing number of borewells were drilled into the limestone dominated Sayq Plateau^3^. For all of these oases Luedeling and Buerkert^11^ had reported an estimated 17% increase in the irrigation requirement from 218.800 m^3^ (1978) to 256.377 m^3^ (2005).

As reported by Gebauer et al.^9^, Luedeling et al.^10^ and Al-Rawahi et al.^3^ agricultural terraces in the four high-altitude oases (1640–1950 m a.s.l.) are typically planted with Mediterranean and temperate climate perennial species such as pomegranate (*Punica granatum* L.), peach (*Prunus persica* L.), rose (*Rosa damascena* L.), walnut (*Junglans regia* L.), and in smaller numbers apricot (*Prunus armeniaca* L.), grape (*Vitis vinifera* L), pear (*Pyrus communis* L.), plum (*Prunus domestica* L.), apple (*Malus domestica* Borkh.), papaya (*Carica papaya* L.), guava (*Psidium guajava* L.), and fig (*Ficus carica* L.). Widespread are also fodder crops such as alfalfa (*Medicago sativa* L.), maize (*Zea mays* L.), wheat (*Triticum* spp.), barley (*Hordeum vulgare* L.), oats (*Avena sativa* L.), and the cash crop garlic (*Allium sativum* L.). Also cultivated are sweet potato (*Ipomoea batatas* L.), potato (*Solanum tuberosum* L.), Guinea gras (*Panicum maximum* Jacq.), eggplant (*Solanum melongena* L.), tomato (*Solanum lycopersicum* L.), chili (*Capsicum frutescens* L.), raddish (*Raphanus sativus* L.), pumpkin (*Cucurbita pepo* L.), lablab (*Lablab purpureus* L.), faba bean (*Vicia faba* L.), cabbage (*Brassica oleracea* L.), salad (*Lactuca sativa* L.), parsley (*Petroselinum crispum* (Mill.) Nym.), and carrot (*Daucus carota* L.). A recent increasingly widespread introduction, sponsored by Oman’s Ministry of Agriculture is the drip-irrigated cultivation of olive (*Olea europaea* L.^28^).

In contrast the low altitude oasis of Masayrat ar Ruwajah is characterized by three-storey arrangements of the subtropical species date palm (*Phoenix dactylifera* L.), lime (*Citrus aurantiifolia* L. Swingle), sweet lime (*Citrus limettioides* Tan.), bitter orange (*Citrus aurantium* L.), citron (*Citrus medica* Burm.), orange (*Citrus sinensis* Osbeck), lemon (*Citrus lemon* (L.) Burm. f.), banana (*Musa* spp.), papaya (*Carica papaya* L.), guava (*Psidium guajava* L.), and mango (*Mangifera indica* L.), while annual plants are the same as in the highlands. In 2007 total cultivated terrace areas amounted to 1.7 ha in Al ‘Aqr, 1.4 ha in Al ‘Ayn, 13.5 ha in Ash Sharayjah, 2.1 ha in Qasha, and 3.5 ha in Masayrat ar Ruwajah.

### Oasis mapping

As a reference to determine landuse changes for the decadal period from March 2007 to April 2018 we accessed the detailed maps of Luedeling and Buerkert^11^. Based on satellite data, georeferenced drone images, and extensive surveys conducted in all oases they contained all trees classified by species, crops as well as idle terrace land as of spring/early summer 2007.

Using the same approach terraced land was re-mapped in spring 2018 whereby data processing was done with the open source software QGIS 2.18 ‘Las Palmas’ (www.qgis.org). As satellite data we used an image of 20 March 2018 (Maxar Technologies) from the Google Earth Pro platform and a 4-band Pan-Sharpened WorldView-3 image of 1 February 2017, both with a spatial resolution of 0.3 m. Data were processed in the geodetic system WGS 84 / UTM zone 40 N with EPSG code 32640.

For the acquisition of ground-truth data, all 3355 field plots of the oases were visited and individually examined for land use and crop species occurrence. The agricultural management of the plots was divided into five different landuse categories, previously selected by Al-Rawahi et al.^24^: (a) “woody plants and crops” = Field cultivation with perennial woody tree and shrub species associated with underplanted annual crops in an agroforestry system, (b) “Woody plants only” = Field cultivation with perennial tree and shrub of species without underplanting of annual crops, (c) “Crops only” = Field cultivation of annual crops without tree and shrub species, (d) "Fallow land” = No crops but intact terrace walls without eroded soil and existing traces of field cultivation such as dead plant remains and soil cultivation, and (e) “Abandonment” = No crops and defective terrace walls with or without eroded soil. To compare the changes in vegetation and landuse between 2007 and 2018, data were used that were generated by Gebauer et al.^9^, Luedeling and Buerkert^11^, Luedeling et al.^10^ and Al-Rawahi et al.^24^.

**Figure 3:**
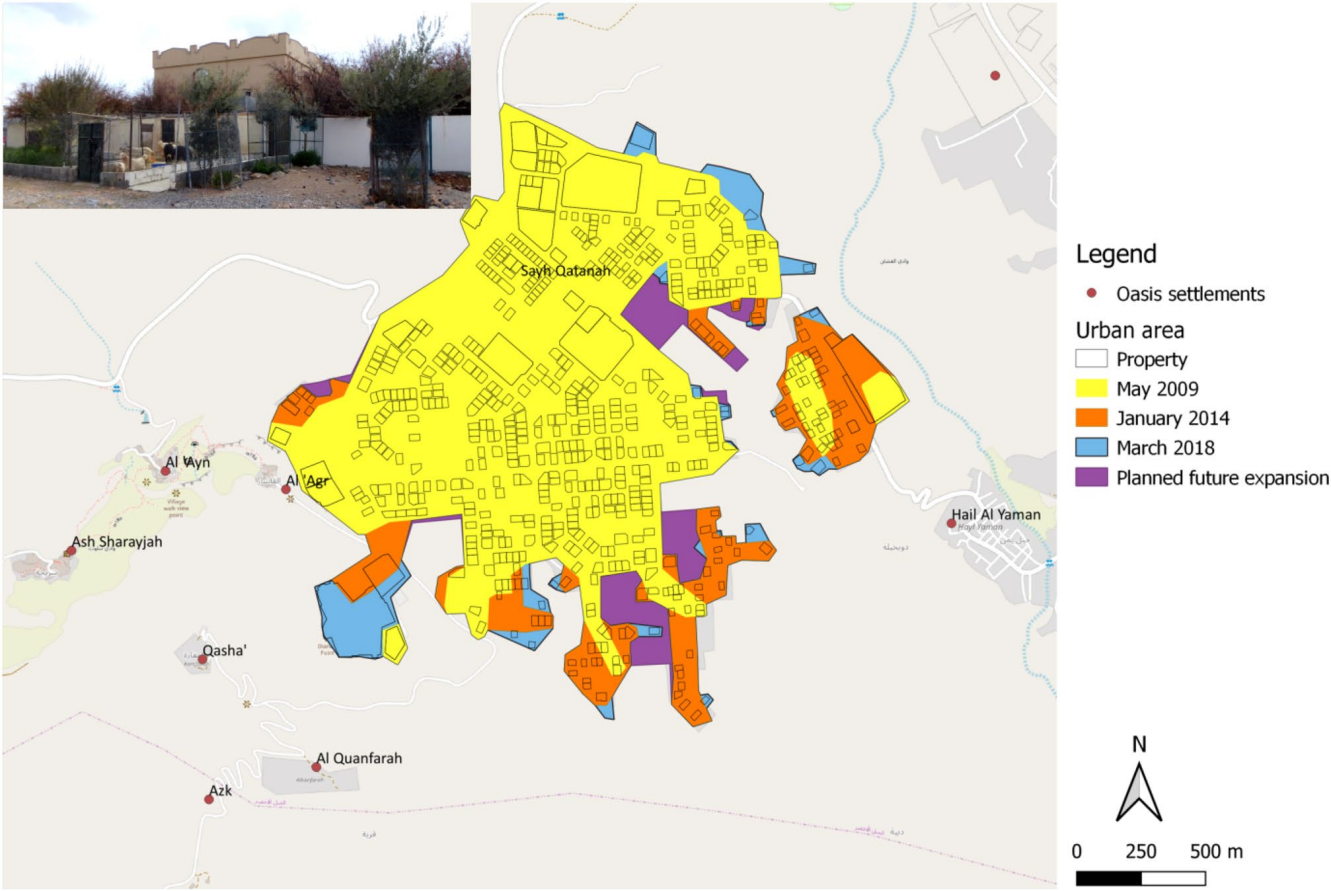
Physical expansion of the town of Sayh Qatanah (2000 m a.s.l.) on the Sayq Plateau of Al Jabal Al Akhdar (Oman) in 2009, 2014 and 2018. The image on the upper left shows a typical private house with a homegarden and vegetable plots plus olive trees outside the walls. Data also show the projected expansion in the coming years based on official town planning (Source: Map Ministry of Housing, Muscat, Oman, unpublished).

The irrigated areas in Sayh Qatanah comprise homegardens located within the property walls and are thus largely hidden from the public. Frequently, only the tree tops give some insights into parts of their structure. Additionally some houses have also gardens outside the property walls which are often secured against the access of free roaming goats and sheep by a wire mesh fence. Most individual accessible perennials were olives or, seldom, ornamental trees (Fig. 3). Based on the satellite images, agricultural and non-agricultural areas, green areas, and building structures were drawn as polygons using a supervised classification of the 2018 satellite images in the QGIS 2.18 ‘Las Palmas’ software (www.qgis.org) and marked with an identification number (ID).

For analysis we selected 25 individual properties at random using a point raster across the town of Sayh Qatanah. The residents were asked for permission to access their homegardens. Whenever a visit was denied or the residents were not present, the closest neighbor was visited to study the structure of this homegarden and to inquire about main crops, irrigation practices, and the origin of irrigation water. The amount of irrigation was estimated by giving details of gallons per month or the amount of money spent each month to irrigate the garden. Based on a fixed water price per gallon the amount of irrigation water per garden and month could be calculated. In all irrigated homegardens outside of the properties cultivated plants were identified and their abundance recorded.

### Determination of landuse changes

To quantify changes in landuse between the two study periods we first compared the total cultivated area using GIS. Subsequently, the actively used land area was calculated by subtracting the area of the landuse category ‘Abandoned’ from the total area. Assuming that fallow land may only be temporarily uncultivated, this landuse category was counted as active landuse. Due to the fluctuating landuse in the study region^11, 24^, the relative distribution of landuse categories was calculated and presented in relation to the total oasis area, including abandoned land for each oasis system separately.

The census of perennial crop species was based on the survey of all terrace plots in the oases and in the settlement of Qanfarah. The census data for Al ‘Ayn, Al ‘Aqr, Ash Sharayjah, Qasha’ and Masayrat ar Ruwajah was evaluated together as well as separately for each oasis. Vegetation changes were determined based on the individual dominance (D_N_) according to Smith and Smith^29^ for the years 2007 and 2018 as follows:

$${D}_{N}=\frac{{N}_{A}}{{N}_{S}}\times 100,$$

*D*_*N*_ = Individual dominance, *N*_*A*_ = Number of individuals of a given species, *N*_*S*_ = Sum of abundance of all species.

Furthermore, the Sørensen coefficient of similarity^30^ was calculated using EstimateS (http://viceroy.eeb.uconn.edu/EstimateS/) to determine the temporal changes in the ligneous population of individual oases gardens from 2007 to 2018 and spatial differences in ligneous species admixtures between oases in either 2007 or 2018.

## Results

### Sayh Qatanah

Since 1978 the town of Sayh Qatanah has experienced a strong physical expansion, initially driven by the building of secondary houses by families from the oases below. This was increasingly followed by population transfer, family growth, tourism facilities, and general expansion of urban infrastructure. The number of developed plots within the town area rose from 276 in 2009 to 534 in 2018 (+ 90%). During the same period the total plot area increased from 41.6 ha to 73.5 ha (+ 77%). This lead to an increase in the urban area from 206 ha in 2009 by 24 ha in 2014 (+ 13.6%^3^) to 252 ha in 2018 (+ 8%). At the current rate of growth, the planned urban space of 268 ha will be reached by 2023, likely followed by densification of the built-up area (Fig. 3). To the east of the city centre a new settlement of 8.6 ha has been established, which, in addition to the typical residential buildings and home gardens, contains a new mosque and an olive grove of 0.7 ha.

**Figure 4:**
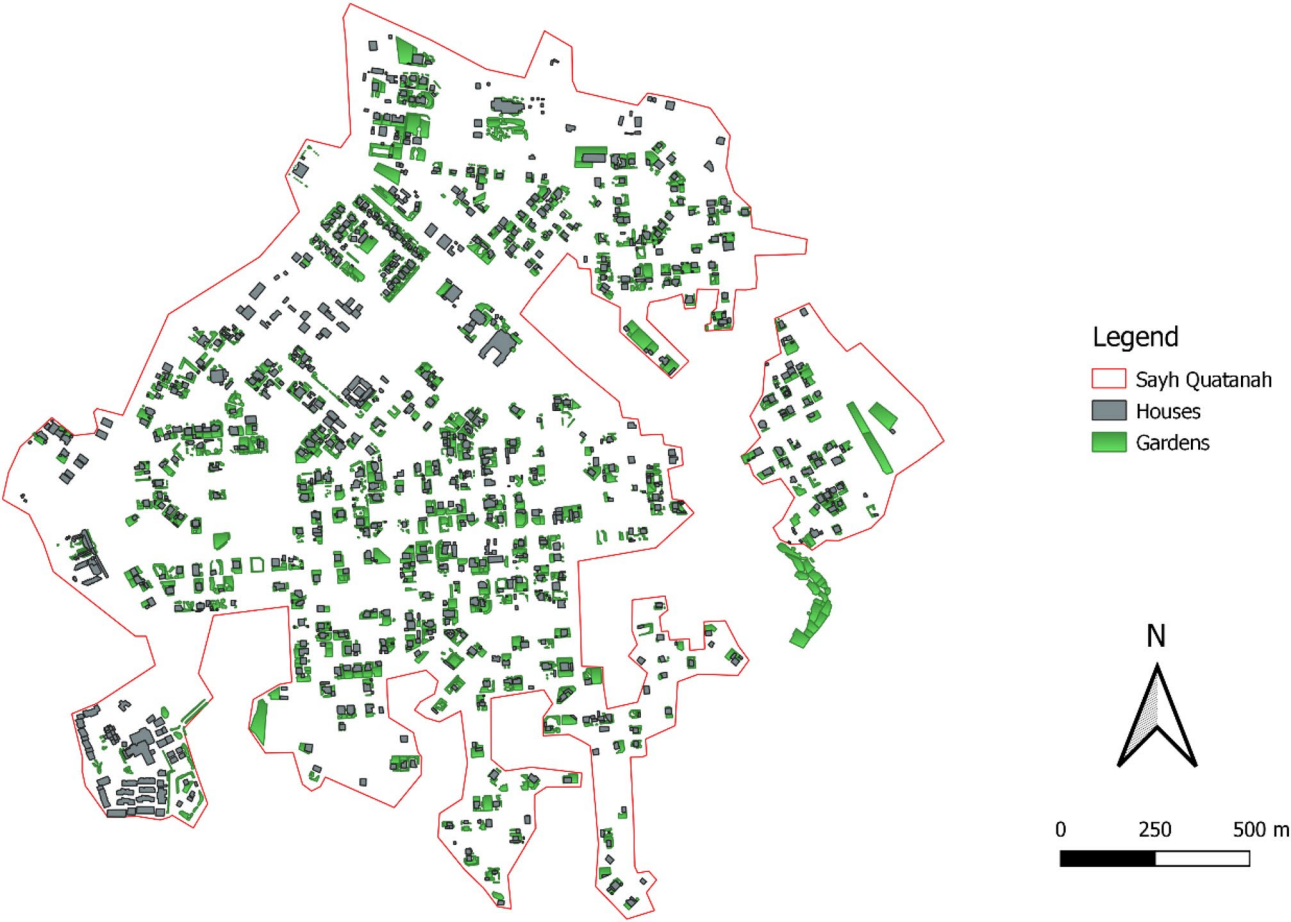
Map of Sayh Qatanah (2000 m a.s.l., Al Jabal Al Akhdar, northern Oman) with all buildings and irrigated areas (gardens) in April 2018.

In 2018 the town’s 56.3 ha non-governmental land comprised 19.3 ha private green spaces, 15.2 ha private buildings, and 4.0 ha public green areas. The total irrigated area thus amounted to 23.3 ha (Fig. 4). The size of individual homegardens ranged between 7 and 3590 m^2^ with an average of 368 m^2^. Some homegardens were partly outside the property wall and contained fruit trees and annual crops. In total 33 perennial and annual plant species of 16 families were identified (Table 1). Abundance was highest for pomegranate, olive, rose bushes, and vine, but also peach, apricot, pear, and fig trees were encountered. Garlic was cultivated in 14 of the 25 homegardens studied, followed by onion, maize, and some fodder barley.

**Table 1:** Species occurrence in the homegardens of the town Sayh Qatanah (Al Jabal Al Akhdar, northern Oman) in 25 randomly selected households.

English name	Latin name	Family	Total no.	Garden no.
Pomegranate	*Punica granatum* L.	Punicaceae	551	25
Olive	*Olea europaea* L.	Oleaceae	262	23
Rose	*Rosa damascena* Mill.	Rosaceae	196	18
Vine	*Vitis vinifera* L.	Vitaceae	88	20
Peach	*Prunus persica* L.	Rosaceae	68	21
Apricot	*Prunus armeniaca* L.	Rosaceae	45	19
Fig	*Ficus carica* L.	Moraceae	45	19
Pear	*Pyrus communis* L.	Rosaceae	36	19
Plum	*Prunus domestica* L.	Rosaceae	34	14
Apple	*Malus domestica* Borkh.	Rosaceae	23	12
Sweet lime	*Citrus limettioides* L.	Rutaceae	22	9
Guava	*Psidium guajava* L.	Myrtaceae	18	10
Banana	*Musa *ssp.	Musaceae	16	6
Citron	*Citrus medica* L.	Rutaceae	16	8
Papaya	*Carica papaya* L.	Caricaceae	14	6
Walnut	*Juglans regia* L.	Juglanda-ceae	13	8
Lime	*Citrus aurantiifolia* (L.) Swingle	Rutaceae	10	7
Mulberry	*Morus nigra* L.	Moraceae	8	7
Kaki	*Diospyros kaki* L	Ebenaceae	2	1
Cherry	*Prunus avium*	Rosaceae	2	1
Lychee	*Litschi chinensis* Sonn.	Sapindaceae	2	1
Bitter orange	*Citrus aurantium* L.	Rutaceae	1	1
Sapodilla	*Manilkara zapota*	Sapotaceae	1	1
Raspberry	*Rubus sectio Rubus*	Rosaceae	1	1
Grapefruit	*Citrus* × *paradise*	Rutaceae	1	1
Almond	*Prunus dulcis *(Mill.) D. A	Rosaceae	1	1
Mango	*Mangifera indica* L.	Anacardia-ceae	1	1
Orange	*Citrus sinensis* (L.) Osbeck	Rutaceae	1	1
Garlic	*Allium sativum* L. var.* sativum*	Liliaceae	–	14
Barley	*Hordeum vulgare* L.	Gramineae	–	2
Maize	*Zea mays* L.	Graminae	–	1
Onion	*Allium cepa *L.	Liliaceae	–	1
Strawberry	*Fragaria* L.	Rosaceae	–	1

Our surveys indicated that besides some private cisterns of unknown capacity for rainwater collection, most residents of Sayh Qatanah used tap water from local borewells for irrigation whereby little attention was given to crop-specific water needs. Average monthly water consumption varied from 43 to 213 l m^−2^ (mean 97 l m^−2^ ± 49 SD). This translated to a total irrigation water use in the 19.3 ha private homegardens of 224,652 m^3^ in 2018. Including the public green areas, the annual estimated water consumption of all green areas of the town amounted to 272,054 m^3^.

### Terrace gardens

Excluding the newly created terrace areas southwest of Ash Sharayjah and the information-free plots of Al ‘Ayn, by 2018 the actively used area of all five oasis systems had declined from 20.3 ha in 2007 to 19.9 ha (− 2.0%). Fallow land increased by 3.5%, while the use of non-perennial crops decreased by 1.9%. The share of perennial crops without underplanting decreased by 5.1%. In contrast, the share of land under agroforestry increased by 2.1% (Table 2). The 2018 plant census yielded an N_S_ of 13,739 with 25 different perennial species from 12 families. The 2007 count resulted in 1150 individuals less, with 24 different species from 14 plant families.

**Table 2:** Landuse of terraces in the oases of Wadi Muyadin, Al Jabal Al Akhdar, northern Oman, in 2007 and 2018. Data of 2007 are from Luedeling and Buerkert^11^.

Landuse class	Area (ha)		$\Delta$ Area (ha)	Change (%)
	2007	2018		
Woody plants + crops	4.9	5.5	0.6	12.1
Woody plants only	12.5	11.0	− 1.4	− 11.5
Crops only	1.3	0.8	− 0.5	− 41.5
Fallow	1.6	2.5	0.9	62.1
Abandonment	7.7	8.1	0.4	5.3
Sum	28.0	28.0		
Actively used land	20.3	19.9	− 0.4	− 2.0

In 2007 D_N_ was highest for pomegranate (51%), rose (21%), date (9%), true lime (5%), peach (4%), and banana (3%). By 2018, D_N_ increased for pomegranate (52%) and rose (28%), but decreased for date (7%), banana (2%), lime (1%), and peach (1%). The establishment of drip-irrigated olive yielded a D_N_ of 4% in 2018, while this crop was non-existent in 2007. Over the past decade olive has thus become the third most common crop species in the study region.

In 2018 the information-free plots of Al ‘Ayn had a similar composition than the other ones, with the three most common species being pomegranate (51%), rose (27%), and olive (6%). Also the newly established Ash Sharayjah terraces were dominated by pomegranate (38%), rose (28%), and olive (23%).

From 2007 to 2018 the N_A_ of most species declined. Sapodilla, pigeon pea, almond, prickly pear (*Opuntia vulgaris* Mill.) and lemon were no longer recorded in the oases. Instead, prickly pear was identified on the newly created terrace areas of Ash Sharayjah and a young almond tree was spotted in Al ‘Ayn. In addition, a sorb tree (*Sorbus domestica* L.) was discovered in Al ‘Ayn. The stand of pome fruits such as apple and pear decreased by 89% and 86%, respectively, and stone fruits recorded a similar decline. The N_A_ of apricots decreased by 88%, while the decline of peaches was 71% and of plums 64%. Bitter orange, true lime, orange, and Palestinian lime were decimated by 91%, 71%, 63%, and 22%, respectively, while date and banana stocks decreased by 14% and 16%. In contrast, the N_A_ of pomegranate increased by 11% and of rose by 50%.

#### Al ‘Aqr

**Figure 5:**
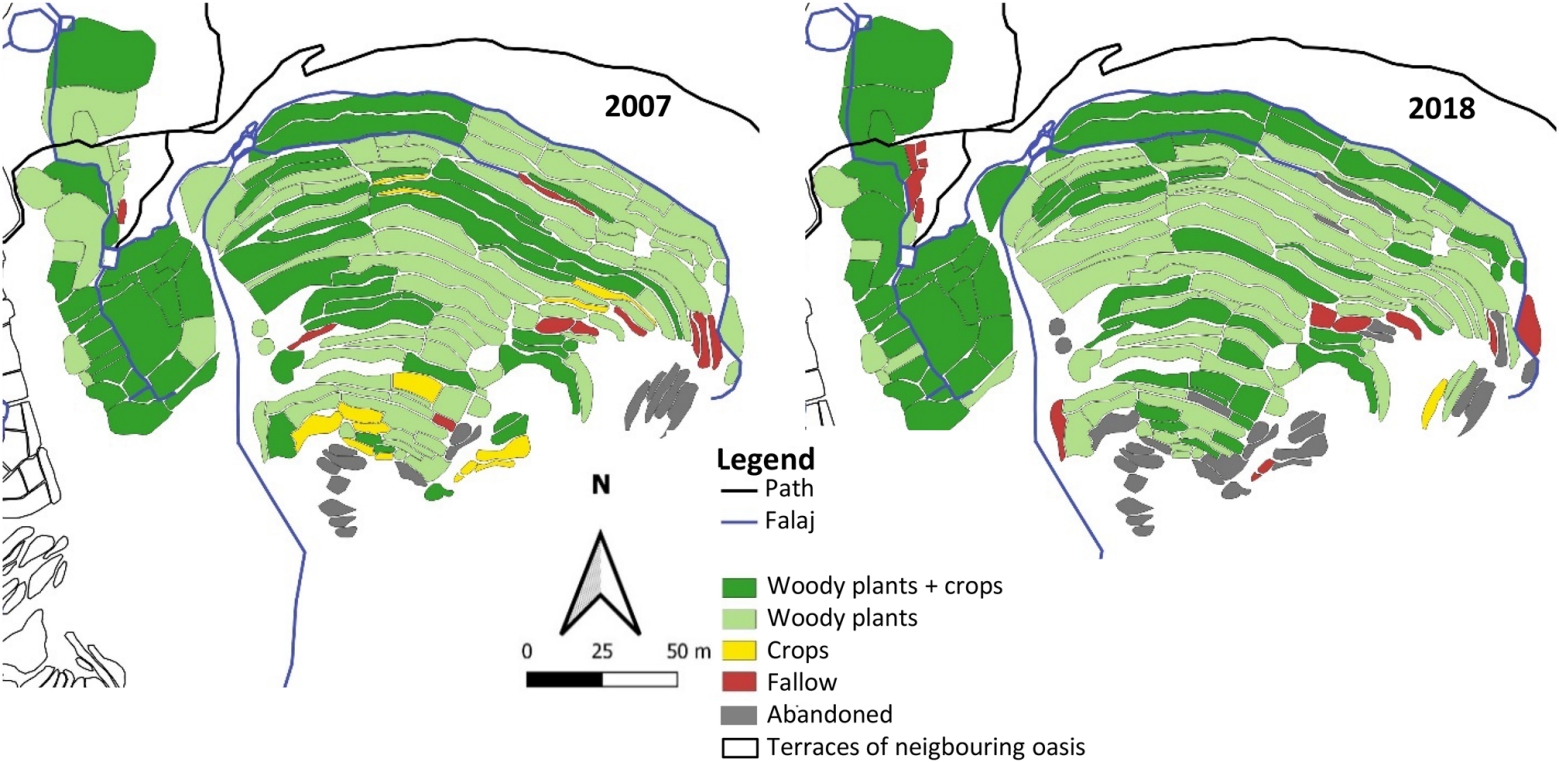
Landuse map of the oasis Al ‘Aqr (1,950 m a.s.l.) in Wadi Muaydin (Al Jabal Al Akhdar, Oman) in 2007 and 2018.

At a constant total terraced area of 1.7 ha the actively used land declined by 3.4% (Fig. 5). Thereof the proportion of agroforestry systems increased by 3.8%, woody plant alone areas declined by 4.8% and annual crops by 3.0%, and fallows increased by 0.8%. Pomegranate and rose were the dominant species in both years (Fig. 5). While the D_N_ of pomegranate decreased from 63.6 to 58.0%, that of rose increased from 22.8 to 39.4%. Whereas the D_N_ of peach fell from 4.5 to 1.6% and bitter orange, orange, lemon, pear and plum completely disappeared, barley, maize, eggplant, and Rhodes grass (*Chloris gayana* Kunth) continued to be cultivated on the terrace areas.

#### Al ‘Ayn

**Figure 6:**
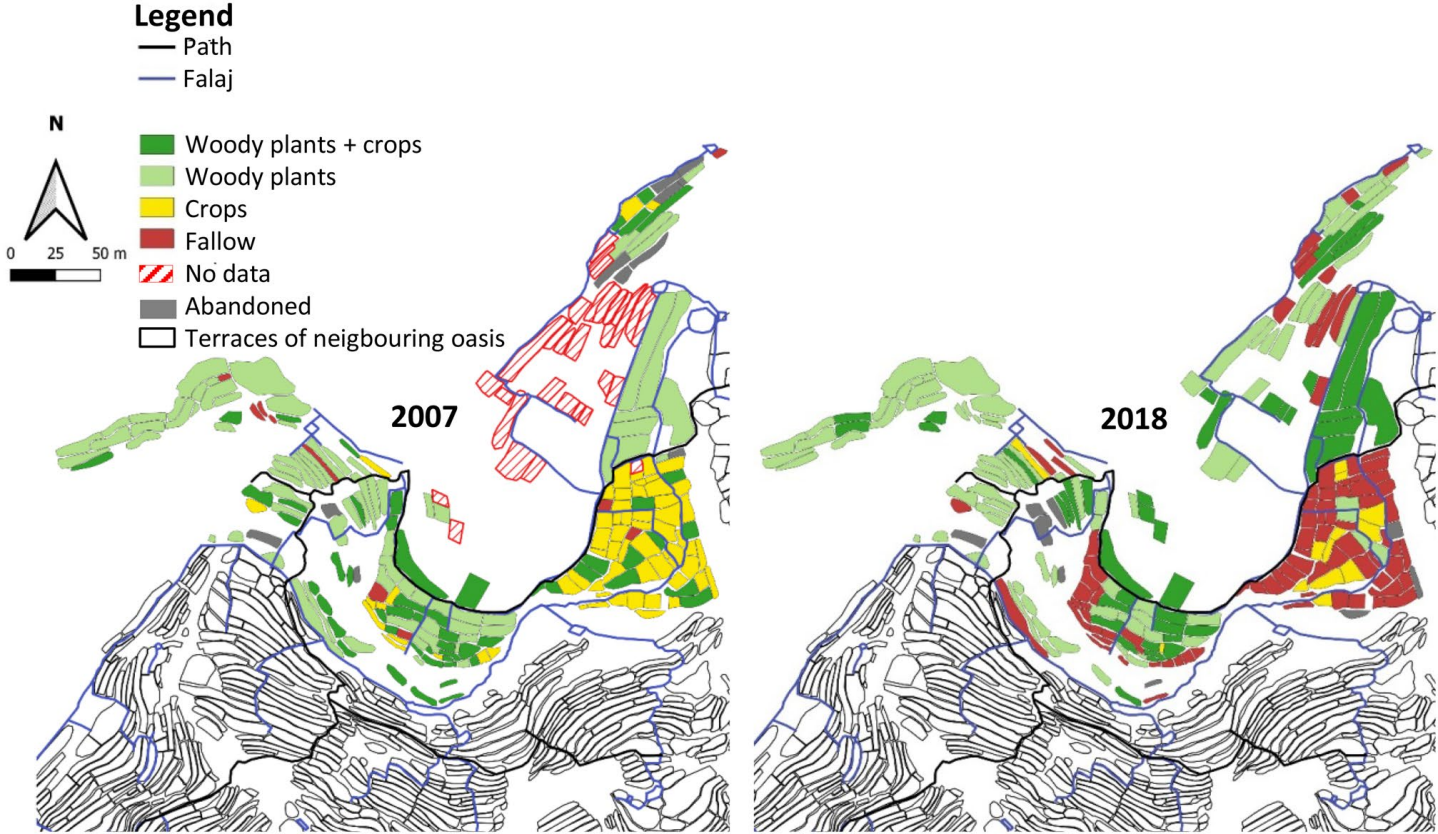
Landuse map of the oasis Al ‘Ayn (1900 m a.s.l.) in Wadi Muaydin (Al Jabal Al Akhdar, Oman) in 2007 and 2018.

Also Al ‘Ayn’s total terraced area of 1.9 ha remained constant. For the 2007 investigation period, information on landuse of ~ 0.3 ha was missing. This was taken into account in the data on relative landuse changes by not considering information-free plots from 2007 which in 2018 contained 20.5% agroforestry systems, 52.0% woody plants only, 1.4% annual crops, and 26.1% fallow land (Fig. 6, Appendix 1).

During the decadal study period the active cultivation area of Al ‘Ayn declined by 0.2%. Areas with agroforestry systems were expanded by 9.3%, while the use of woody plants only recorded a decline of 16.4%, fallow land increased by 25.1%, and the annual cropping area declined by 18.1% (Fig. 6).

Between 2007 and 2018, the D_N_ of rose increased from 54.6 to 61.8% and of pomegranate from 28.2 to 30.5%. The D_N_ of peach decreased from 4.3 to 2.1%, and of papaya, lime, and apricot to less than 2.0%. In contrast to 2007, no records of apple and lemon were obtained in 2018. However, barley, garlic, onion, sweet potato, sorghum, and oats continued to be cultivated.

#### Ash Sharayjah

**Figure 7:**
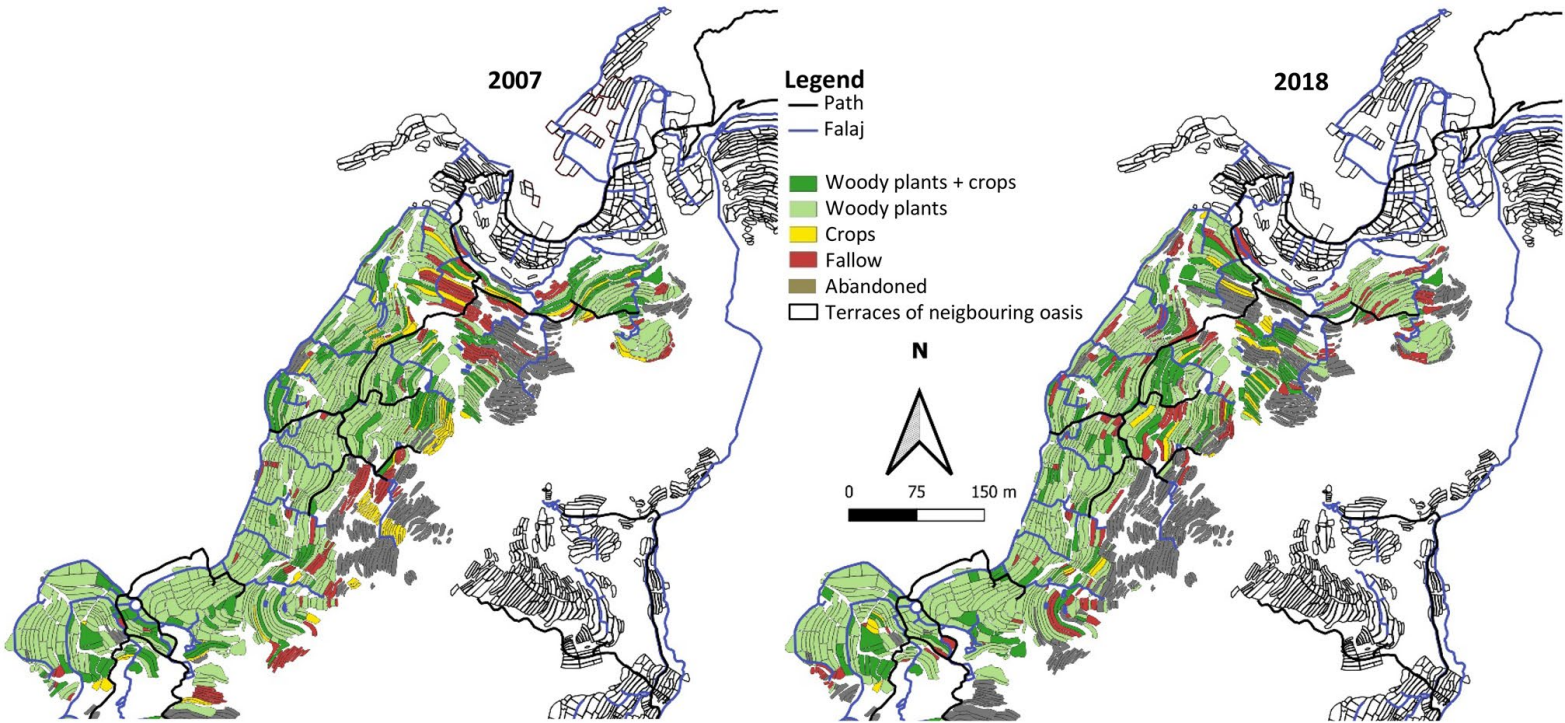
Landuse map of the oasis Ash Sharayjah (1900 m a.s.l.) in Wadi Muaydin (Al Jabal Al Akhdar, Oman) in 2007 and 2018.

In 2007 Ash Sharayjah’s total area was 15.2 ha to which, by 2018, 1.7 ha of newly developed farmland were added and included in our digital mapping (Fig. 7, Appendix 2). For the determination of relative area changes, however, these newly established terraces areas were not taken into account. During the transformation decade the agriculturally used area of Ash Sharayjah decreased by 4.5%. The total area with agroforestry systems increased by 0.2%, woody plants only declined by 4.5%, areas with annual crops decreased by 2.1% and fallow fields expanded by 3.1% (Fig. 7).

Until 2018 the D_N_ of roses increased from 21.4 to 26.7%, while if fell for pomegranate from 63.9 to 61.7%, for true lime from 5.6 to 0.9%, and for apricot and peach it declined to < 2.0%. Also the D_N_ of date (− 83%), apricot (− 81%), bitter orange (− 80%), true lime (− 78%), pear (− 78%), peach (− 65%), walnut (− 44%), and grape (− 44%) declined compared to 2007, while only a single specimen of the bitter orange was sighted in 2018 and prickly pear was only found in the newly established terraces. The latter, located in the southwest of Ash Sharayjah, were built with cement and some of them had a large field structure. In contrast to the traditional terraces they were often drip-irrigated and planted to perennials such as pomegranate (38%), rose (28%), and olive (23%). Overall agroforestry systems occupied 9% and annual crops 1.4% on this new land.

#### Qasha’

**Figure 8:**
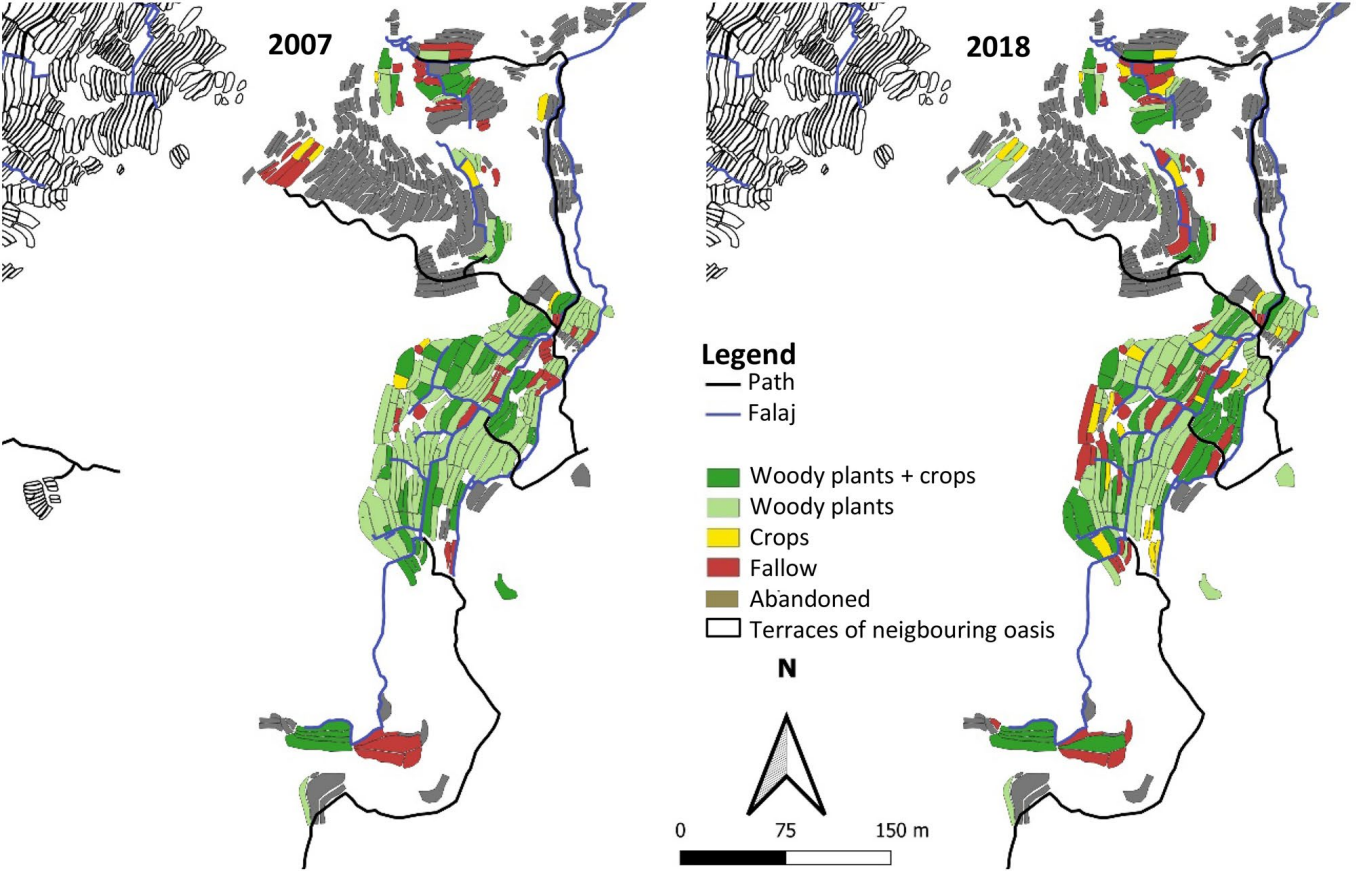
Landuse map of the oasis Qasha’ (1640 m a.s.l.) in Wadi Muaydin (Al Jabal Al Akhdar, Oman) in 2007 and 2018.

Qasha' covered a total area of 4.9 ha. In 2018, 56% of the terrace fields were abandoned while the agriculturally used area increased from 1.8 to 1.9 ha (+ 4.7%). Areas with agroforestry systems increased by 6.1%, those with woody plants declined by 6.3%, annual crops increased by 1.9% and fallow by 1.3% (Fig. 8). The D_N_ of pomegranate increased from 56 to 74%, of rose from 9.1 to 29.7%, and of banana from 5.7 to 5.9%. In contrast, D_N_ of peach declined from 12.1 to 0.9% and D_N_ of grape and walnut were each < 2.0%.

Decreases in N_A_ were noted for peach (− 92%), walnut (− 86%), apricot (− 85%), date (− 70%), lime (− 47%), sweet lime (− 43%), fig (− 38%), and grape (− 33%), while the N_A_ of guava (+ 450%) and banana (+ 3%) increased. Individuals of lemon, mango, olive, and plum were resettled, whereas mash apple, bitter orange, orange, papaya and pears were not further cultivated. In 2018 on the terraced areas the annual species maize, garlic, tomato, oats, onion, Rhodes grass, potato, coriander, carrot, and pepper were cultivated.

#### Masayrat ar Ruwajah

**Figure 9:**
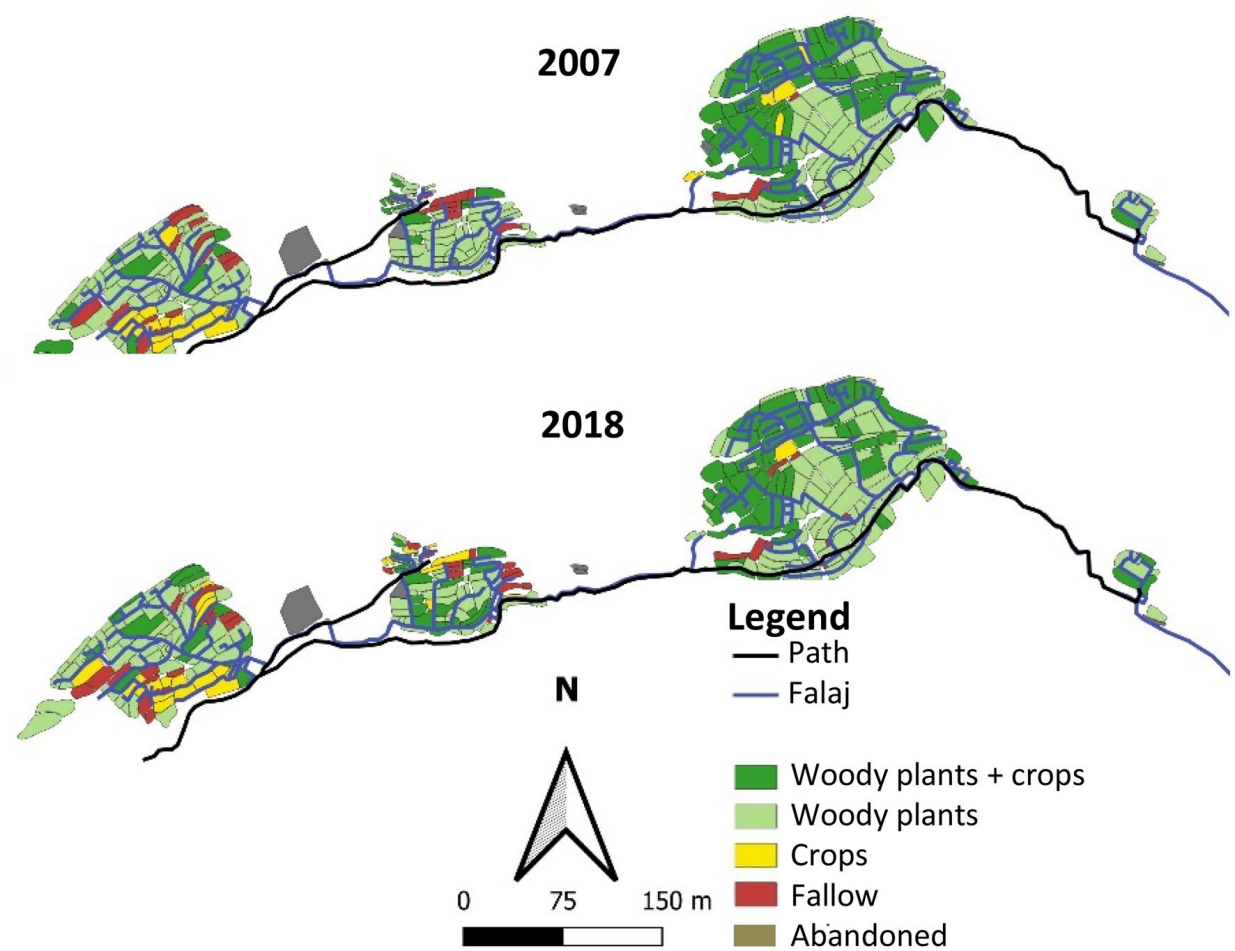
Landuse map of the oasis Masayrat ar Ruwajah (1030 m a.s.l.) in Wadi Muaydin (Al Jabal Al Akhdar, Oman) in 2007 and 2018.

The total area of Masayrat ar Ruwajah (3.4 ha) remained unchanged over the past decade, while the agriculturally used terraces increased by 0.3% from 3.05 ha to 3.06 ha. Relative landuse by agroforestry systems increased by 1.3%, areas with woody plants experienced a slight decline (0.2%), while annual crops and fallow areas remained largely unchanged (+ 0.1% each; Fig. 9).

With respect to the N_S_ of all perennial crops the fruit stock in Masayrat decreased by 7.7%, while date, banana, and true lime remained the most common species across time. The D_N_ of date increased from 67 to 70%, although its N_A_ declined by 15%. The D_N_ of banana fell from 14.5 to 14.1% and that of lime from 8.3 to 5.2%. Declines in N_A_ were also noted for peach (− 84%), lime (− 48%), grape (− 25%), and banana (− 19%) while the abundance of guava and Palestinian lime remained unchanged. In contrast the N_A_ of papaya (+ 54%), fig (+ 73%), and mango (+ 167%) increased. In contrast to 2007, no apricot and lemon were recorded and Rhodes grass was the most widespread annual crop followed by barley, sorghum, maize, and oats as fodder crops on smaller areas. Alfalfa and vegetables such as onion, garlic, chili, and eggplant were rarely cultivated.

#### Qanfarah

**Figure 10:**
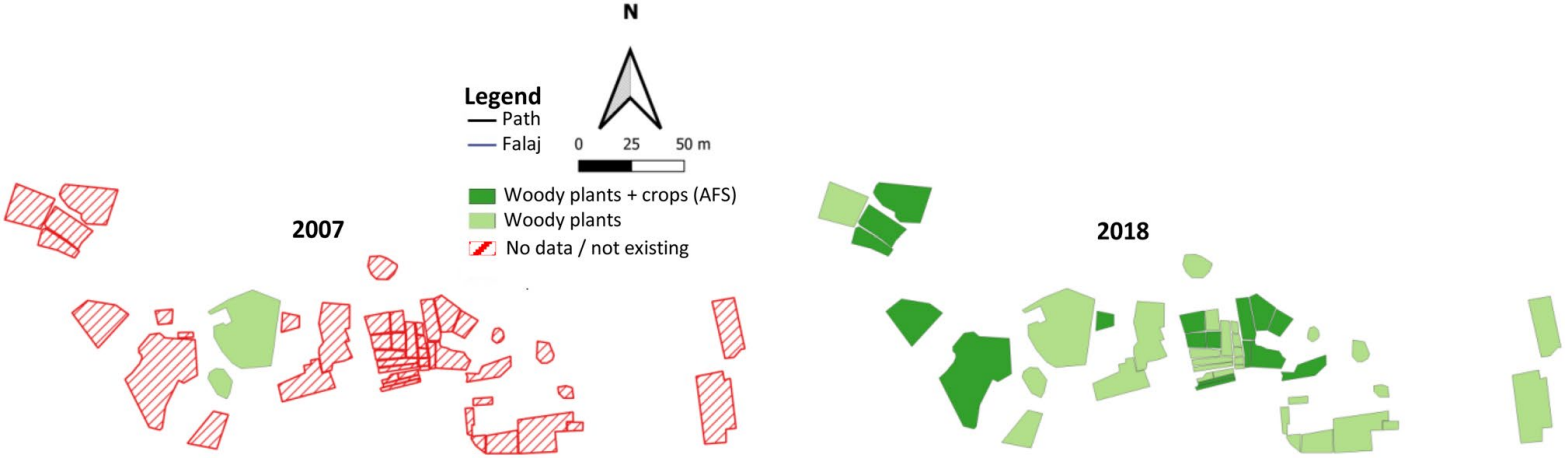
Landuse map of the oasis Qanfarah (1700 m a.s.l.) in Wadi Muaydin (Al Jabal Al Akhdar, Oman) in 2007 and 2018.

In 2018 landuse of the largely newly established terraced areas of Qanfarah was recorded for the first time. Therefore no comparison with the 2007 data is possible. The agricultural area amounted to 0.7 ha largely composed of homegardens (Fig. 10). They are surrounded by high walls, a protection against unauthorized entry and browsing livestock. We recorded a total of 29 species from 20 plant families. The highest D_N_ had pomegranate (34%), followed by rose (29%), olive (6%), banana (5%), apricot (3%), guava (3%), and grape (3%). Also recorded were lemon, Palestinian lime, fig, and papaya as well as corn, barley, garlic, eggplant, helmet bean, potato, chili, strawberry, and sugar cane.

#### Spatio-temporal variation of ligneous species diversity

The Sørensen coefficient of similarity, used to compare the ligneous plant species in a given oases between 2007 and 2018, indicated that changes in species composition were lowest in Masayrat ar Ruwajah, where the coefficient exceeded 0.90 (Table 3). In the high altitude oases of Al ‘Ayn, Al ‘Aqr and Ash Sharayjah, similarity of species between 2007 and 2018 was also high with coefficients ≥ 0.8 in all cases. The most important temporal changes occurred in Qasha’ where the coefficient was only 0.72.

**Table 3:**
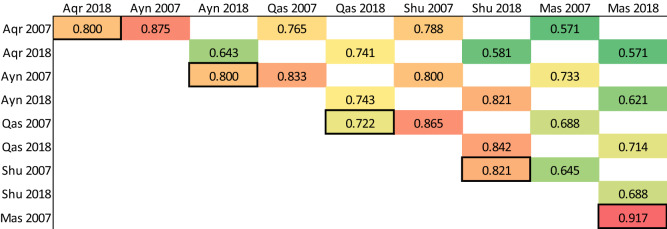
Sørensen coefficient of similarity comparing the diversity of fruit trees in 2007 and 2018 within the oases* of Wadi Muaydin (2007 *versus* 2018, boxed values) and across oases (2007 and 2018, respectively).

*Oases are abbreviated with the three first letters of their main name.

For Qanfarah only data of 2018 was available, therefore it is not included here.

Due to the altitudinal gradient, species cultivated in Masayrat ar Ruwajah have always differed from those of the other oases and respective coefficients did not change considerably from 2007 to 2018. Ligneous plant stands in Qasha’ and Ash Sharayjah were similar in 2007 (0.87) and 2018 (0.84). The same applied to the ligneous species cultivated in Al ‘Ayn and Ash Sharayjah (2007: 0.80; 2018: 0.82). In contrast, ligneous stands in Al ‘Ayn and Al ‘Aqr were similar in 2007 (0.88), but differed considerably (0.64) in 2018. Species changes were most important between Al ‘Aqr and Ash Sharayjah where the coefficient dropped from 0.79 in 2007 to 0.58 in 2018.

## Discussion

As reported by Al-Kalbani et al.^25^ during the last decade most inhabitants of Al ’Ayn, Al ’Aqr, Ash Sharayjah, Qasha’, and Masayrat ar Ruwajah have left their old houses in the oases of Wadi Muaydin and moved to the newly established town of Sayh Qatanah. This spatial shift of population and related irrigated agricultural land led to a raise of annual freshwater consumption from borewells drilled to 300 m depth in Sayh Qatanah from 310,200 m^3^ in 2007 to 685,154 m^3^ in 2018 (unpublished data of Al Jabal Al Akhdar Water Authority). This was complemented by the daily amount of 420 m^3^ (153,300 m^3^/year) for Sayh Qatanah and 1 m^3^ (365 m^3^/year) for Ash Surayjah of recycled wastewater from the newly established Sewage Treatment Plants making irrigation water available according to WHO standards. In 2019, after completion of the Jabal Akhdar mountain pipeline, 563,959 m^3^ desalinized sea water were pumped in from Barka (at 70 km aerial distance across the rugged Hajar Mountains and a 2000 m elevation difference).

Our data support our initial hypothesis of a major landuse change that occurred during the last decade in the oases of Wadi Muaydin, which especially shows in the results for individual species’ dominance but less in the temporal variation (2007 *versus* 2018) of the Sørensen coefficient of similarity. Particularly noteworthy is the strong decline of temperate plant species and the increasing occurrence (D_N_) of rose, pomegranate, and olive. The latter has become the fourth most common crop in the study region. This is striking as no olive trees were observed in the study region in 2007^9^. The strongly increasing D_N_ of pomegranate and rose, as a consequence of the rising economic importance of both crops in the region^28^, underlines the increasingly (tourist-)sales oriented nature of agriculture in the upper oases of Wadi Muaydin^31, 32^. The intensification of pomegranate and rose cultivation may also reflect the effects of rising temperatures on Al Jabal Al Akhdar: Already a decade ago Luedeling et al.^10^, corrobated by a recent study of Buerkert et al.^33^, predicted changes in the crop composition of the study region as a consequence of global warming. They hypothesized that decreasing winter chilling may lead to decreasing yields of temperate fruit and nut tree species, which would force farmers to shift to alternative crop species. Our data shows that during the last decade, particularly in the high-altitude oases, a strong decline in the N_A_ of pome and stone fruit occurred, species that typically require a certain amount of hours with low temperatures (“Chill hours” or “Chill portions”) for flowering and fruit initiation^9^. Their decline may have become yield-limiting for some of the temperate species. According to the Sørensen coefficient of similarity, the greatest changes in ligneous plant stands occurred at mid-altitude, namely in Qasha’, while the composition of ligneous stands remained very stable in Masayrat ar Ruwajah and showed only moderate temporal change in the high-altitude oases. This result is substantiated by the fact that during field work farmers in Qasha’ repeatedly reported crop failure in pomegranate and other chill-sensitive crops, which seem to support the claims of Luedeling et al.^10^ and merits further study. As far as species composition changes among the high-altitude oases in the ten-year study period are concerned, the greatest decrease (− 21%) in the Sørensen coefficient of similarity occurred between Al ‘Aqr and Ash Sharayjah. Since the coefficient remained relatively stable for the comparison of Ash Sharayjah with Al ‘Ayn and with Qasha’, respectively, this indicates that the greatest changes in species composition of ligneous plants stands occurred in Al ‘Aqr, even though neither the temporal comparison (Sørensen coefficient 2007 *versus* 2018) for this oasis nor the indicators proposed by Smith and Smith^29^ reflect this.

Our terrace mapping shows that the abundance of pomegranate has increased significantly over the last decade as have rose and guava, particularly in Qasha’ and in Ash Sharayjah. This may be due to the fact that the surface of these two oases is, with 4.9 ha and 15.2 ha, much larger than that of Al ‘Aqr (1.7 ha). From 2007 to 2018 the number of ligneous individuals in Al ‘Aqr increased by + 23% as compared to + 20% and + 31% in Qasha’ and in Ash Sharayjah, while the number of ligneous species declined by − 33% and − 11% in the two former and increased by + 17% in the latter oasis. These results demonstrate that the use of several diversity indicators is useful to unravel species changes in ecologically similar yet spatially different locations^34^. A shift to rose, pomegranate, and drip-irrigated cultivation of olive was also observed on the newly planted terraces of Ash Sharayjah. This likely reflects the combined effect of growing upper class tourist markets in Sayh Qatanah offering reliable marketing opportunities as well as the effects of water scarcity, mainly in the hot summer months.

Our data of species change on the terraced fields may also indicate the effects of a rising competition for water, severely enhanced by the substantial expansion of the town of Sayh Qatanah with its rapidly growing housing areas, irrigated homegardens, and public greenery. This may, together with increasing scarcity of skilled labour, explain expanding terrace areas under fallow or abandonment. Across the Wadi Muaydin watershed the latter rose from 4.8 ha in 2007 to 10.6 ha in 2018. At the same time the area of terraces cropped with only annual species and without perennial crops fell from 3.5 ha in 2005^11^ to 0.8 ha in 2018. These changes result in potential water savings and higher water use efficiency on irrigated plots which may be one reason for the increased establishment of agroforestry systems as well as areas with woody plants only, in Wadi Muaydin.

## Conclusions

The transformation of oasis agriculture in our study area resembles processes in many Arabian countries. On Al Jabal Al Akhdar during the past decade a changing lifestyle of its rapidly growing population has led to the relocation of many remote terraces gardens of the old oasis systems to new conveniently accessible homegardens in the plateau city of Sayh Qatanah. Their irrigation, higher water use by locals, and the consequences of rising national and international tourism led to excessive groundwater extraction from a single watershed fed by calcareous rocks of low water retention capacity.

Farmers in Wadi Muaydin adapted to the new transformation-related challenges of water and labour scarcity by making changes in land use and crop choice. Consequences were a decrease in local production of water-demanding forage crops towards more water efficient agroforestry systems and increased reliance on food and feed imports from the lowlands and international markets. The limited terrace areas are increasingly used for the market-oriented production of rose water, pomegranates, and confined husbandry of small ruminants based on imported fodder. The urbanization-related decline in opportunities for herded grazing on the mountain ranges is accompanied by a growing separation of the animal and crop husbandry systems. Their integration was over centuries the basis for the sustainable landuse systems in Omani agriculture and shaped the country’s unique Arabian traditions. Physical infrastructure, such as terraces, irrigation canals, and connecting mountain paths can easily be preserved by modern-day brick and cement infrastructure. More important challenges to the continued existence of oasis systems in the Western Hajar Mountains of Oman are labor and water scarcity, loss of traditional knowledge and community structures, climate change, and declining species diversity. Therefore, political and economic support schemes will be required to help preserving these systems as ideotypes of ancient social-ecological land use and cultural heritage of the Arabian Peninsula.

## Figures and Tables

**Figure 11:**
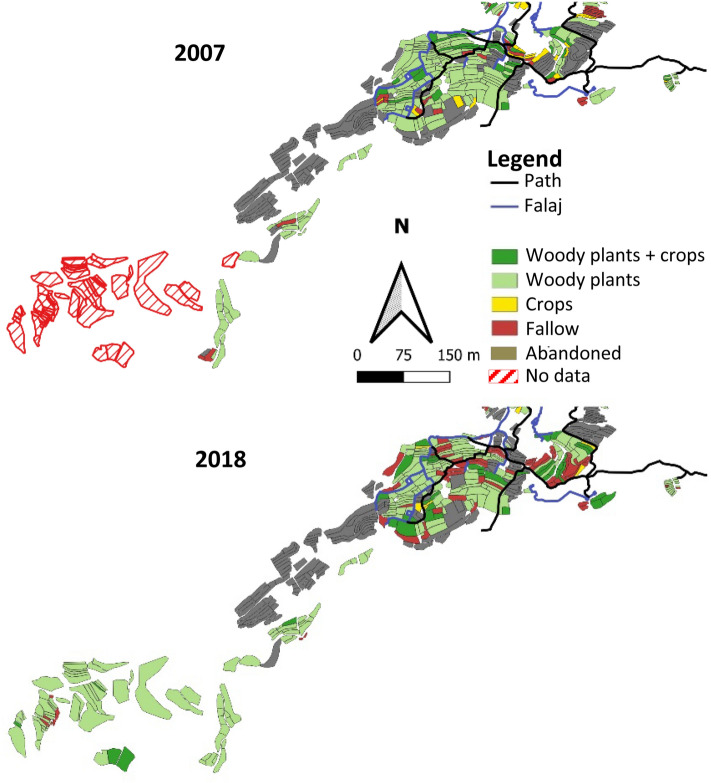
Landuse map of the oasis Ash Sharayjah-South in Wadi Muaydin (Al Jabal Al Akhdar, Oman) in 2007 and 2018.

## Appendix 1

See Table 4.

**Table 4:** Landuse in the terraces of Wadi Muaydin, Al Jabal Al Akhdar, Oman in 2007 and 2018.

	Land use class	Area (m^2^)		∆ Area (m^2^)	Change (%)
		2017	2018		
Oasis of Al ‘Aqr	Woody plants + crops	574	1116	542	94.4
	Woody plants only	560	49	− 511	− 91.3
	Crops only	8055	7250	− 805	− 10.0
	Fallow	7304	7944	640	8.8
	Abandonment	327	461	134	41.0
	Sum	16,820	16,820	0	0
	Actively used land	16,245	15,704	− 541	− 3.3
Oasis of Al 'Ayn	Woody plants + crops	558	585	27	4.8
	Woody plants only	3637	827	− 2810	− 77.3
	Crops only	7595	5039	− 2556	− 33.7
	Fallow	3349	4792	1443	43.1
	Abandonment	366	4262	3896	1064.5
	Sum	15,505	15,505	0	0
	Actively used land	14,947	14,920	− 27	> 0
Oasis of Ash Sharayjah	Woody plants + crops	39,417	44,534	5117	13.0
	Woody plants only	5914	2686	− 3228	− 54.6
	Crops only	77,960	71,052	− 6908	− 8.9
	Fallow	19,261	19,504	243	1.3
	Abandonment	9560	14,336	4776	50.0
	Sum	152,111	152,111	0	0.0
	Actively used land	112,694	107,578	− 5116	− 4.5
Oasis of Quasha'	Woody plants + crops	27,688	26,148	− 1540	− 5.6
	Woody plants only	756	1708	952	125.8
	Crops only	10,510	7405	− 3105	− 29.5
	Fallow	7207	10,229	3022	41.9
	Abandonment	3369	4040	671	19.9
	Sum	49,530	49,530	0	0
	Actively used land	21,843	23,383	1540	7.1
Oasis of Masayrat ar Ruwajah	Woody plants + crops	1057	960	− 97	− 9.2
	Woody plants only	2171	2354	183	8.4
	Crops only	17,786	17,042	− 744	− 4.2
	Fallow	10,493	10,923	430	4.1
	Abandonment	2007	2235	228	11.4
	Sum	33,515	33,515	0	0
	Actively used land	32,458	32,555	97	0.3

## Appendix 2

See Fig. 11.
